# Mechanical Ventilator-Associated Pneumonia in the COVID-19 Pandemic Era: A Critical Challenge in the Intensive Care Units

**DOI:** 10.3390/antibiotics14010028

**Published:** 2025-01-03

**Authors:** Mircea Stoian, Adina Andone, Sergiu Rareș Bândilă, Danusia Onișor, Sergiu Ștefan Laszlo, Gabriela Lupu, Alina Danielescu, Dragoș-Florin Baba, Anca Meda Văsieșiu, Andrei Manea, Adina Stoian

**Affiliations:** 1 Department of Anesthesiology and Intensive Care, George Emil Palade University of Medicine, Pharmacy, Sciences and Technology of Târgu Mures, 540139 Targu Mures, Romania; mircea.stoian@umfst.ro; 2 Intensive Care Unit, Mures Clinical County Hospital, Street Gheorghe Marinescu No 1, 540103 Targu Mures, Romania; laszlo.sergiu.stefan@gmail.com (S.Ș.L.); lupu.gabriela@stud18.umfst.ro (G.L.); alinka942@yahoo.com (A.D.); 3 Gastroenterology Department, George Emil Palade University of Medicine, Pharmacy, Sciences and Technology of Târgu Mures, 540142 Targu Mures, Romania; adina.roman@umfst.ro (A.A.); danusia.onisor@umfst.ro (D.O.); 4 Orthopedic Surgery and Traumatology Service, Marina Baixa Hospital, Av. Alcade En Jaume Botella Mayor, 03570 Villajoyosa, Spain; bandila_ser@gva.es; 5 Department of Cell and Molecular Biology, George Emil Palade University of Medicine, Pharmacy, Science and Technology of Targu Mures, 540142 Targu Mures, Romania; dragos-florin.baba@umfst.ro; 6 Infectious Disease, George Emil Palade University of Medicine, Pharmacy, Sciences and Technology of Targu Mures, 540136 Targu Mures, Romania; anca-meda.vasiesiu@umfst.ro; 7 Doctoral School of Medicine and Pharmacy, George Emil Palade University of Medicine, Pharmacy, Science and Technology of Targu Mures, 540139 Targu Mures, Romania; 8 Department of Radiology, Clinical Emergency County Hospital of Targu Mures, 540136 Targu Mures, Romania; 9 Department of Pathophysiology, George Emil Palade University of Medicine, Pharmacy, Sciences and Technology of Targu Mures, 540136 Targu Mures, Romania; adina.stoian@umfst.ro

**Keywords:** mechanical ventilation, nosocomial infections, viral and bacterial co-infections, ventilator-associated pneumonia, multidrug-resistance

## Abstract

**Background/Objectives:** Ventilator-associated pneumonia (VAP) is the most common nosocomial infection encountered in the intensive care unit (ICU) and is associated with prolonged hospitalization and increased mortality. We evaluated the causative pathogens involved and their resistance to the major classes of antibiotics in patients with VAP and assessed the differences between patients with and without coronavirus disease 2019 (COVID-19). **Materials and Methods**: This study was a single-center, cross-sectional, retrospective analysis involving 122 patients who were hospitalized in the ICU of Târgu Mureș County Clinical Hospital from 1 April 2021, to 1 April 2023. This study compares patients with VAP in COVID-19 and non-COVID-19 groups, examining the clinical progression, duration of ventilation and hospitalization, mortality, pathogen distribution, and the emergence of multidrug-resistant strains. **Results:** A length of stay in the ICU exceeding 11.5 days was associated with the development of multidrug-resistant (MDR) infections (AUC: 0.708, *p* < 0.001). Similarly, a duration of MV exceeding 196 h was associated with MDR acquisition (AUC: 0.695, *p* = 0.002). Additionally, a Clinical Pulmonary Infection Score (CPIS) greater than 5 was associated with MDR development (AUC: 0.854, *p* < 0.001) in the whole group of patients. The most commonly isolated strains were *Acinetobacter* spp., *Pseudomonas* spp., Klebsiella spp., and *Staphylococcus aureus*. Among non-COVID-19 patients, there was a notably higher frequency of MDR *Acinetobacter baumannii*. A bacterial resistance to carbapenems was found in *Acinetobacter* spp. (51.6%), *Klebsiella* spp. (22.6%), and *Pseudomonas* spp. (25.8%). **Conclusions:** COVID-19 patients experienced longer ventilation, higher mortality, and an increased risk of developing MDR. Carbapenem resistance was universal in *Acinetobacter* spp. and *Klebsiella pneumoniae*, whereas resistance in *Pseudomonas aeruginosa* was more prevalent among non-COVID-19 patients. The Clinical Pulmonary Infection Score (CPIS) strongly correlates with developing MDR pathogens in both patient groups.

## 1. Introduction

Much data have been published in the literature regarding VAP in patients diagnosed with Severe Acute Respiratory Syndrome Coronavirus 2 (SARS-CoV2), but these findings cannot be generalized, which is why the understanding of the microbiological profile in different regions is crucial [1]. In Romania, the first case of COVID-19 was identified on 15 February 2020, and since then, almost 3.5 million cases of COVID-19 and almost 70,000 deaths have been reported [2].

Pneumonia may be complicated by respiratory failure, requiring supplemental oxygen or invasive (IMV) or non-invasive (NIV) mechanical ventilation [3]. Although considered a “necessary evil”, mechanical ventilation (MV) is a life-saving technique. Despite all efforts to adapt ventilation to physiological breathing, it can cause numerous adverse effects, both in the lungs and in other peripheral organs [4].

The main purpose of MV in the ICU is to protect the airway in patients with low levels of consciousness, brain trauma, stroke, or drug overdose, and those undergoing anesthesia (perioperative period). It can also address hypercapnic respiratory failure caused by respiratory diseases, diseases of the chest wall or respiratory muscles, and hypoxemic respiratory failure [5,6,7,8,9].

VAP is pneumonia that occurs 48 h or more after the initiation of MV, most commonly through an endotracheal tube or tracheostomy [10]. It results from the invasion of the lower respiratory tract and lung parenchyma by microorganisms acquired during MV [10]. Clinical signs of VAP include purulent tracheal secretions, fever, and respiratory distress [10]. VAP, along with hospital-acquired pneumonia, represents the most common hospital-acquired (nosocomial) infection and is associated with significant morbidity and mortality risks [11,12].

VAP affects between 40% and 55% of patients requiring IMV for more than 48 h, with variations depending on the country, the type of intensive care, and the criteria used for diagnosis [13]. Approximately 10% of patients who receive MV develop VAP, leading to prolonged ventilation durations, extended hospital stays, and increased healthcare costs [14].

Pneumonia can be classified based on its development into early-onset VAP (≤4 days after initiation of MV), which is primarily caused by susceptible, non-MDR pathogens, and late-onset VAP (>4 days after initiation of MV), which is predominantly caused by MDR organisms [15,16,17]. Risk factors for MDR VAP include prolonged antibiotic therapy, septic shock, acute respiratory distress syndrome, and acute renal replacement therapy [16,17].

The microorganisms involved in VAP include bacterial, viral, or fungal pathogens, with an increased prevalence of those from the ESKAPE group (*Enterococcus faecium*, *Staphylococcus aureus*, *Klebsiella pneumoniae*, *Acinetobacter baumannii*, *Pseudomonas aeruginosa*, and *Enterobacter* spp.). These pathogens present a global threat because of their resistance to most of the available antibiotics [18], thus VAP is considered a severe ICU-acquired infection. Treatment typically involves broad-spectrum antibiotics to cover all likely pathogens until specific microorganisms are isolated [16]. Extensive antibiotic use increases the risk of developing MDR microorganisms, which can lead to adverse outcomes and higher mortality rates [19,20,21].

Recent studies have shown that COVID-19 patients who require IMV are at a higher risk of developing VAP, leading to worse outcomes and putting pressure on the healthcare system [22,23].

In our article, we aimed to analyze the differences between patients with VAP in the COVID-19 group and those in the non-COVID-19 group. We focused on several aspects: clinical progression, the duration of ventilation and hospitalization, mortality, the distribution of pathogens involved in VAP among the two patient groups, and the emergence of MDR strains.

## 2. Results

### 2.1. Patient Characteristics. Demographic and Clinical Aspects

The Mureș County Clinical Hospital, Târgu Mureș, is a tertiary care center from Romania that has 1182 beds, with 44 beds allocated to the ICU, 23 beds to the General Intensive Care Unit (polyvalent), and 21 beds to the Post-Anesthesia Intermediate Intensive Care Unit (PACU).

Between 1 April 2021, and 1 April 2023, a total of 11,648 patients were admitted to the Mureș County Clinical Hospital. In the ICU, excluding post-anesthesia care units, 3341 critical patients were admitted, with 912 requiring MV. After applying the inclusion and exclusion criteria, 122 (13.4%) were included in the study and diagnosed with VAP (Table 1).

The main clinical and demographic data of the patients included in this study are presented in Table 2. The sex distribution of the 122 patients included in the study was 75 (61.5%) men and 47 (38.5%) women.

Out of the 122 patients, 53 (43.4%) were diagnosed with a COVID-19 infection on admission. The mean age of the patients in the COVID-19 group was 70.04 ± 11.48, while in the non-COVID-19 group, the mean age was 66.58 ± 11.79, which was not significantly different (*p* = 0.107; Table 3).

There were no significant differences in the length of stay in the ICU between the COVID-19 group and the non-COVID-19 group (*p* = 0.242; Table 3). However, patients in the COVID-19 group were ventilated for longer in the ICU (*p* = 0.001; Table 3).

The assessment of previous contact with the hospital or other healthcare institutions and the risk of intra-hospital infection according to the Carmeli score indicated that 64 out of the 122 patients (52.5%) presented an increased risk.

The assessment of procalcitonin levels (PCT) indicated that 16 (13.1%) patients had procalcitonin values above 2 ng/mL (normal values are below 0.5 ng/mL) upon admission, but this number doubled to 31 (25.4%) after 48 h. All 122 patients who exhibited clinical signs suggestive of VAP underwent a series of radiological investigations. The first investigation was essential to confirm the diagnosis of VAP, and subsequent radiological assessments were conducted to monitor the progression of each case. Additionally, some patients underwent both conventional radiological examinations and chest CT scans. In a dynamic radiological assessment, 37 patients underwent thoracic CT scans, and 19 of them (51.4%) exhibited indicative evolutionary radiological changes. Additionally, 106 patients received conventional radiological evaluations, with 54 of them (51%) showing similar evolutionary radiological changes.

There was a significant statistical difference between the Carmeli score at admission between the two groups (*p* = 0.005). There was also a substantial difference between the number of comorbidities between the two groups, with non-COVID-19 patients having more comorbidities than COVID-19 patients (*p* < 0.001). Statistically significant differences between the two groups were also noticed regarding invasive devices (central venous catheter *p* = 0.012 and urinary catheters < 0.001) and evolutive pulmonary lesions of patients that underwent CT scans (*p* = 0.049).

### 2.2. Laboratory Data

The ICU hospitalization characteristics and laboratory results are detailed in Table 3.

The leukocyte count did not show significant differences between the two groups at admission (*p* = 0.764), 48 h (*p* = 0.248), or 72 h (*p* = 0.054). Fibrinogen and C-reactive protein (CRP) levels did not show significant differences between the groups. The neutrophil/lymphocyte ratio showed significant differences between groups at 48 (17.8 vs. 12.02, *p* = 0.016) and 72 h (24.31 vs. 10.13, *p* < 0.001); in both cases, COVID-19 patients had higher values. Procalcitonin (PCT) tested negative at admission in COVID-19 patients (*p* = 0.006, chi-square test) and 48 h (*p* = 0.012), suggesting the absence of bacterial infection or co-infection at admission.

### 2.3. Pathogens Involved in VAP Resistance to Antibiotics

Table 4 displays the relative frequencies of microorganisms isolated from the bronchial aspirates of patients. Among COVID-19-positive patients, *Acinetobacter* spp. is the most frequently isolated microorganism, accounting for 39.29% of cases, followed by *Pseudomonas* spp. at 21.43% and *Klebsiella* spp. at 14.29%. Non-COVID-19 patients show a similar pattern in the frequency of these pathogens; however, they exhibit a higher relative frequency of multidrug-resistant (MDR) *Acinetobacter baumannii* compared to the COVID-19 group. All *Acinetobacter* spp. and *Klebsiella pneumonia* strains that are MDR were resistant to carbapenems in both groups. Among the *Pseudomonas aeruginosa* MDR strains identified in COVID-19-positive patients, 50% were also carbapenem-resistant. In contrast, all *Pseudomonas aeruginosa* MDR strains found in COVID-19-negative patients were resistant to carbapenems.

The species most frequently encountered were *Acinetobacter* spp., *Klebsiella* spp., and *Pseudomonas* spp. Their resistance to commonly used antibiotics in the ICU was assessed.

In our analysis of antibiotic resistance among patients diagnosed with VAP, regardless of their COVID-19 status, we found the following results:

- Carbapenem Resistance: 51.6% was attributed to *Acinetobacter* spp., 25.8% to *Pseudomonas* spp., and 22.6% to *Klebsiella* spp..
- Resistance to Third-Generation Cephalosporins: 66.7% was attributed to *Pseudomonas* spp., followed by *Acinetobacter* spp. and *Klebsiella* spp., both at 16.7%.
- Fluoroquinolone Resistance: 47.8% was attributed to *Acinetobacter* spp., 43.5% to *Pseudomonas* spp., and 8.7% to *Klebsiella* spp..
- Aminoglycoside Resistance: 48.1% was attributed to *Acinetobacter* spp., 33.3% to *Pseudomonas* spp., and 18.5% to *Klebsiella* spp..

These findings highlight the significant levels of antibiotic resistance among the bacteria involved.

### 2.4. Antimicrobial Treatment

The most often used antibiotic drugs were carbapenems (26 %), third-generation cephalosporins (24.9%), fluoroquinolones (13.9%), and polymyxins (10.4%). Overall, 87% of the patients received combinations of at least two antibiotics, whereas only 13% received monotherapy. No patient included in the study received treatment with first-generation cephalosporins or trimethoprim/sulfamethoxazole.

Antibiotic therapy was administered in intermittent individualized infusions for each patient at regular intervals. Combinations of two or more antibiotics were necessary until the causative microorganisms were isolated or in the case of infection with several bacteria or fungi (Table 5).

### 2.5. Severity Scores in the ICU

**Figure 1:**
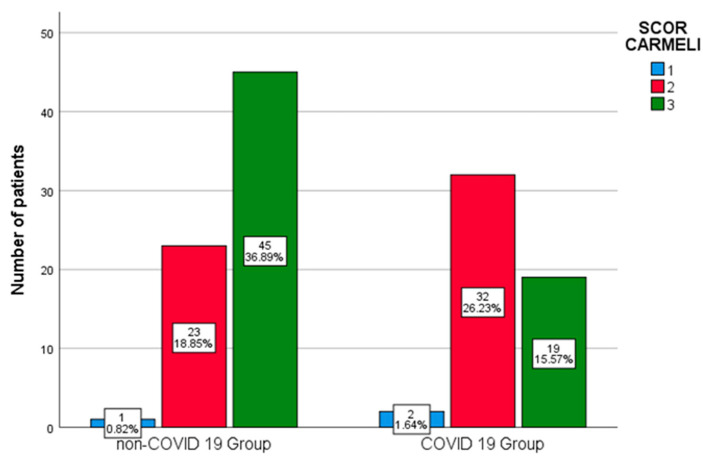
Carmeli score between the two groups.

An analysis of the Carmeli score data indicated that most of the non-COVID-19 patients had a Carmeli score of 3 upon admission, whereas those with COVID-19 had a Carmeli score of 2 (*p* = 0.005; Figure 1).

**Figure 2:**
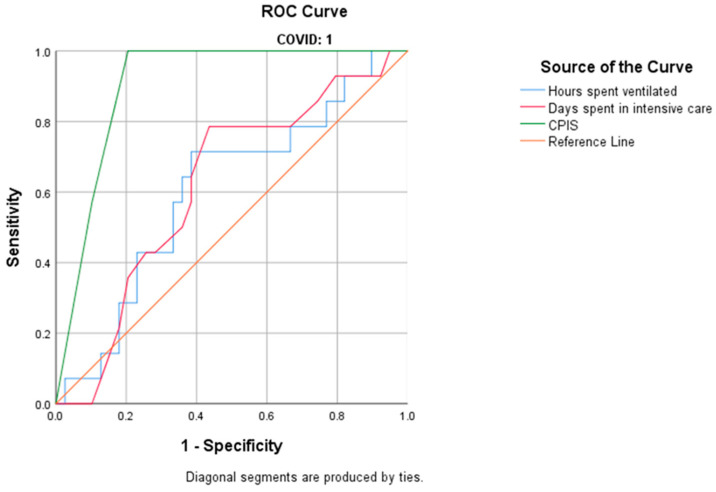
ROC curve of the univariate analysis of COVID 19 patients.

The median (Q1–Q3) Acute Physiology and Chronic Health Disease Classification System (APACHE) II score upon admission to the ICU was 24 (17.50–27.50) for patients in the COVID-19 group and 17 (14–25) for the non-COVID-19 group. The median (Q1–Q3) Sequential Organ Failure Assessment (SOFA) score was 10 (8.00–12.25) in the COVID-19 group and 10 (6.00–12.50) in the non-COVID-19 group. The Clinical Pulmonary Infection Score (CPIS) in the group of COVID-19 patients was statistically significantly correlated with the development of MDR pathogens in COVID-19 patients (*p* < 0.001). By analyzing the receiver operating characteristic (ROC) curve, we determined that the area under the curve (AUC) was 0.905. Based on the curve, a CPIS above 5 is associated with 100% sensitivity and 79.5% specificity in the development of MDR bacteria in the COVID-19 patient group (Figure 2; Table 6).

**Figure 3:**
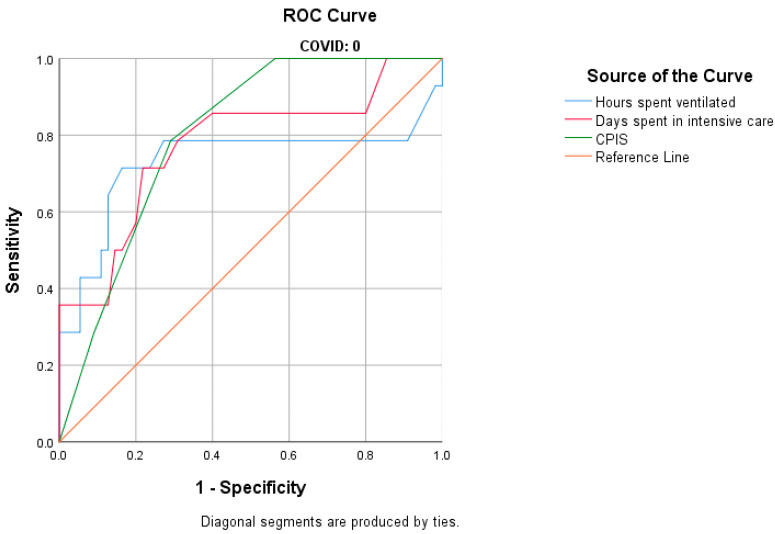
ROC curve of the univariate analysis of non-COVID-19 patients.

The CPIS was also correlated with the development of MDR bacteria in non-COVID-19 patients (*p* < 0.001). When analyzing the ROC curve, we found an AUC of 0.800. We observed that a CPIS above 5 is associated with a sensitivity of 78.6% and a specificity of 70.9% in predicting the development of MDR bacteria (Figure 3; Table 6).

### 2.6. Invasive Devices

The analysis of invasive devices indicated that almost all COVID-19 patients had a central venous catheter, being more favorable to have one than non-COVID-19 patients (*p* = 0.012, Fisher exact test). COVID-19 patients had urinary catheterization at admission at a significantly lower percentage (13.1% vs. 46.7%) compared to the non-COVID-19 group (*p* < 0.001, chi-square test). In terms of ventilation, COVID-19 patients underwent both IMV and NIV during hospitalization, while non-COVID-19 patients were invasively ventilated more often (*p* < 0.014, chi-square test).

### 2.7. VAP/MDR Relationship

We performed univariate analysis to assess the diagnostic performance of multi-drug resistance, the dependent variable, about each of the independent variables (days spent in intensive care, hours spent on ventilation, and CPIS). The analysis was conducted for each of the three patient groups: COVID-positive, COVID-negative, and all patients combined. Each variable was examined individually through a univariate analysis. We utilized a ROC curve to evaluate the diagnostic capability of each variable. The optimal cut-off value was determined using the Youden Index, which maximizes the sum of sensitivity and specificity for each analysis.

The relationship between the duration of ventilation, length of stay in the ICU, CPIS, and the development of MDR strains, in all patients and separated into groups, is presented in Table 6.

**Figure 4:**
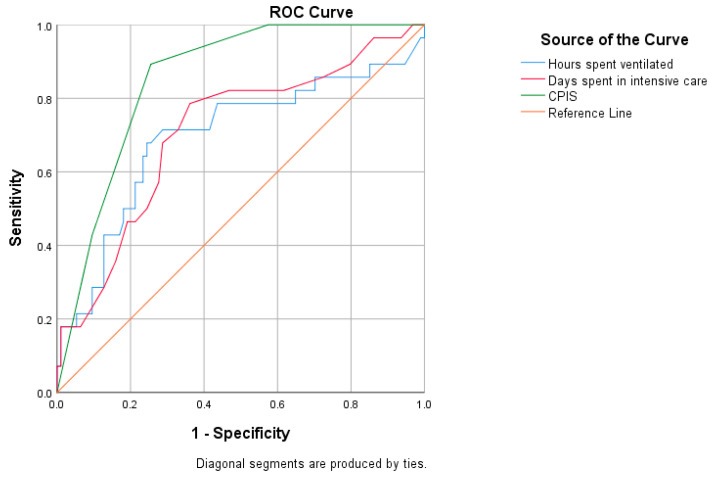
ROC curve of the univariate analysis of all patients.

The ROC analysis indicated a positive correlation between hospitalization duration and MDR pathogen development. In patients hospitalized for 11.5 days, the sensitivity was 78.6% and the specificity was 63.8%. The AOC was 0.708 with a *p*-value of 0.001 (Figure 4).

The ROC analysis of the duration of ventilation in hours and the development of MDR pathogens found that a ventilation lasting over 196 h was associated with the development of MDR pathogens. The sensitivity of this association is 78.6% and the specificity is 56.4%. The AOC was 0.695, with a *p*-value of 0.002 (Figure 4).

### 2.8. Mortality

COVID-19 patients had a higher risk of death than COVID-19-negative patients (*p* = 0.002, chi-square test). By comparing mortality using the chi-square test, we observed that patients who underwent IMV had a higher risk of death compared to patients ventilated with NIV or mixed ventilation (*p* < 0.001). The risk of death was associated with prolonged stays in the ICU (*p* = 0.017), a prolonged duration of ventilation (*p* = 0.001), leukocytosis at 48 and 72 h (*p* < 0.001), and an increase in neutrophils at 48 and 72 h (*p* < 0.001). Patients who showed evolutive radiological changes on chest radiographs (*p* = 0.003, chi-square test) or CT scans (*p* = 0.007, Fisher exact test) had a higher risk of death than those without changes (Table 7).

## 3. Discussion

Data from the literature indicate that VAP is a common cause of infections acquired in the ICU [16,24,25]. Despite efforts to prevent VAP, it remains the most common infection in ICU patients with MV.

The overall incidence of VAP in our study was 13.4%, based on the number of patients admitted and ventilated in the ICU. Out of the patients in the study who developed VAP, 56.6% were from the non-COVID group and 43.4% were from the COVID-19 group. Some authors reported an incidence of VAP of 45.4% during the pandemic [26], compared to 9.2% in the pre-pandemic period in Portugal (2016–2017) [25]. In France, 8% of patients admitted to the ICU in 2016 developed hospital-acquired pneumonia, with the majority (88.7%) diagnosed with VAP [27]. Endotracheal tube intubation (ETT) is the primary risk factor for VAP, as it is associated with over 95% of pneumonia cases in the ICU. The presence of the ETT disrupts the natural protective barriers of the upper airway, creating direct access to the tracheobronchial tree. While the high-volume, low-pressure cuff of the ETT helps reduce aspiration, it does not fully prevent micro aspiration. Additionally, if the cuff pressure drops or the ETT moves, secretions can be pulled into the airway [28,29]. This includes patients with impaired consciousness, trauma, and advanced age, and is influenced by the severity of the underlying disease for which they were admitted to the ICU [30,31].

VAP is suspected based on clinical criteria, with pulmonary radiological changes playing a central role. The presence of a new, persistent pulmonary infiltrate lasting 48 h or more, or evidence of progressive infiltrates, is considered essential for diagnosis. However, these findings alone are not always sufficient to definitively establish VAP [32]. Postmortem studies indicate that, while these radiological changes combined with other criteria—such as fever, leukocytosis, and purulent secretions—show moderate sensitivity (69%) and specificity (75%), they can be overlooked in certain cases [33]. For instance, in patients with acute respiratory distress syndrome (ARDS), new infiltrates may be difficult to detect radiologically. In these situations, the lack of radiological changes does not eliminate the possibility of VAP, particularly if there are other clinical signs or if blood gas levels worsen. While radiological findings are an important factor, they are not always essential for every case. Additional evaluations are necessary for a precise diagnosis [32]. Howroid et al. described an algorithm for diagnosing VAP, emphasizing the significance of symptoms and clinical characteristics of pulmonary infection, particularly in mechanically ventilated patients with respiratory deterioration. When the CPIS is uncertain because of missing data, clinicians should conduct sputum sampling and chest imaging to confirm the diagnosis [28].

VAP is associated with increased morbidity and mortality, longer hospital stays, and higher healthcare costs, especially when caused by MDR pathogens [24,34,35]. For more than a decade, we have dealt with the spread of MDR bacteria in hospitals and the community [36,37,38].

The crucial question is how accurately VAP cases are reported. Unfortunately, there are no comprehensive reports available to provide a clear picture in Romania. However, European reports on the prevalence of healthcare-associated infections, estimated incidence, and the composite index of antimicrobial resistance in hospitals offer some comparative data. According to these reports for the period 2016–2017, Romania had a prevalence of nosocomial infections of below 4%, while the European mean was 7%, suggesting a possible misreporting of nosocomial infections [13,39,40]. According to reports, Romania ranks third among European countries in terms of performance, following Lithuania and Bulgaria. However, when it comes to treatment resistance for these infections, Romania ranks first in Europe, with a concerning rate of 68.9%, compared to the European mean of 30% [13]. These statistics raise questions about their accuracy and suggest the possibility of underreporting VAP cases.

A systematic review and meta-analysis published in 2020 examined the accuracy of various clinical indicators for diagnosing VAP in critically ill adult patients. The study revealed that none of the indicators—such as fever, leukocytosis, chest radiographs, protected specimen brushing (PSB), bronchoalveolar lavage (BAL) cultures, or endotracheal aspirate (ETA)—demonstrated sufficient sensitivity or specificity to reliably diagnose VAP. All of these parameters, including the CPIS, showed low accuracy when compared to histopathological analysis, which served as reference standards. The findings of this review emphasize the challenges of diagnosing VAP based solely on clinical signs. Relying on these tests may result in misdiagnosis and unnecessary antibiotic use. The study underscores the necessity for more reliable diagnostic tools to enhance clinical decision-making and reduce overtreatment in managing VAP [41]. Upon analyzing the demographic data of VAP patients, male patients were more numerous, as follows: 75 (61.5%) patients were men and 47 (38.5%) were women. This observation was also noted in studies by Shah et al. (2022) on the African-American population [42], by Mergulhão et al. (2024) in a multicenter study in ICUs in Portugal [25], and by Wu et al. (2019) in a meta-analysis that included 20 provinces in China [30].

Patients included in the study had an increased incidence of cardiovascular and renal comorbidities, diabetes, and obesity. Previous research suggests that the outcome of patients with VAP depends on various factors, including patient demographics, comorbidities, socioeconomic conditions, disease severity, specific causative bacteria, antibiotic resistance, and virulence [20,25,35]. All of the patients with VAP in our study underwent MV, especially IMV, and had previous exposure to extended-spectrum antibiotics according to the Carmeli score. Male sex and severity of the underlying disease are independent risk factors leading to the development of VAP, as shown by Wu et al. [30]. Chronic conditions such as coronary artery disease, diabetes, and chronic renal failure have been documented as risk factors for VAP [25,43].

Data from the literature show that the use of IMV is associated with increased morbidity, mortality, and healthcare costs [44], as well as the development of VAP [24]. Tracheal intubation and IMV disrupt the defense mechanisms of the respiratory system and interfere with the protective reflexes of the respiratory system, thereby preventing effective coughing, affecting mucociliary clearance, and promoting micro aspiration of oropharyngeal contents [45]. Most cases of VAP are caused by the micro aspiration of oropharyngeal secretions that accumulate above the cuff of the endotracheal tube or tracheostomy tube [46].

From our data, the patients in the COVID-19 group were mainly on both NIV and IMV, in contrast to patients in the non-COVID-19 group, who were mainly on IMV from the beginning (*p* = 0.014). Data obtained from 2552 immunocompromised patients with acute respiratory failure show that NIV was associated with a significantly lower mortality rate, especially in immunosuppressed patients with human immunodeficiency virus, hematological malignancies, and bone marrow transplantation [47].

The duration of MV is another risk factor associated with VAP, with a cumulative risk of 3% per day during the first week and 2% per day during the second week of ventilation. The highest risk of infection occurs between 8 and 10 days [48].

According to the Carmeli score, 52.5% of the subjects were at an increased risk of antibiotic resistance (Carmeli score of 3). This assessment tool is used in Romania to evaluate the risk of resistance to antibiotic treatment when patients are admitted to health facilities. An analysis of the Carmeli score indicated that non-COVID-19 patients had a Carmeli score of 3 more often than the COVID-19 group upon admission, which suggests previous contacts with health facilities (*p* = 0.005) [2].

Acute or chronic mental disorders, such as anxiety, depression, agitation, or delirium, were present in 17 (13.9%) patients. Experiences in intensive care, critical illness, ventilation, noise, and artificial light could trigger mental disorders, including post-traumatic stress disorder, post-ICU depressive states, and anxiety syndromes. These elements have also been observed in other research [49,50,51]. This observation should be further analyzed in other studies, owing to the significant impact on the quality of life of these patients.

The recommendations for administering oxygen therapy in cases of acute respiratory failure in patients with COVID-19 follow this sequence: oxygen delivered via a mask or nasal cannula, high-flow nasal oxygen, NIV, and IMV [52,53,54].

The duration of ventilation in patients from the COVID-19 group was longer (*p* = 0.001) compared to the non-COVID-19 group. The COVID-19 group was also associated with higher mortality (*p* = 0.001). These data were also observed in other studies [23,55,56].

At 72 h, significant changes were observed with an increase in neutrophils (*p* = 0.017), a decrease in lymphocytes (*p* < 0.001) and a decrease in monocytes (*p* = 0.006) in COVID-19 patients. Other authors have also observed similar modifications in monocytes and lymphocytes in previous studies [57,58]. A decreased lymphocyte count and low serum albumin levels were identified as independent risk factors for 30-day mortality [59]. The neutrophile/lymphocyte ratio showed significant differences between groups at 48 h (*p* = 0.016) and 72 h (*p* < 0.001), which has also been reported in other countries [60,61,62].

The diagnosis of Candida pneumonia has been and continues to be widely debated because of its rarity and the frequent presence of colonization. The only way to prove that VAP is due to *Candida* spp. is to use a lung biopsy with the associated evidence of inflammation. The conclusion that VAP is caused by a species of Candida based only on its presence in respiratory samples and the decision to initiate antifungal therapy is controversial owing to the lack of clinical evidence [49]. The most commonly isolated strains in COVID-19-positive patients were *Acinetobacter* spp. (39.29%), *Pseudomonas* spp. (21.43%), *Klebsiella* spp. (14.29%), and *Staphylococcus aureus* spp. (7.14%). In patients who tested negative for COVID-19, the most frequently isolated strains included *Acinetobacter* spp. (32.34%), *Pseudomonas* spp. (11.76%), *Staphylococcus aureus* (8.82%), and *Klebsiella* spp. (11.76%). *Acinetobacter baumannii* MDR strains were more prevalent in COVID-19-negative patients (29.41% versus 17.86%). *Acinetobacter* spp., *Klebsiella pneumoniae*, *Pseudomonas aeruginosa*, *Staphylococcus aureus*, *Escherichia coli*, and *Candida* spp. were the most widespread causative microorganisms identified in endotracheal aspiration (ETA) samples in the pre-pandemic period in the ICU [63,64]. During the pandemic period, Yasemin et al. (2022) reported that the following microorganisms were isolated in ETA samples: *Acinetobacter baumannii* (54.0%), *Klebsiella pneumoniae* (10.3%), *Pseudomonas aeruginosa* (6.8%), *Enterococcus faecium* (8%), and *Candida* spp. (13.7%) [63]. By far the most frequently isolated microorganism in patients with VAP is *Acinetobacter baumannii* [65]. *Acinetobacter baumannii* is an opportunistic pathogen that has developed multiple antibiotic resistance mechanisms and is responsible for the production of infectious outbreaks, especially in the ICU [66]. *Acinetobacter baumannii* represents a significant threat to immunocompromised patients with chronic diseases such as cardiac, renal, and diabetic, especially in ICUs that have a high exposure to invasive procedures and prolonged antibiotic therapy [67,68]. *Acinetobacter baumannii* easily survives on both wet and dry surfaces. It can colonize the skin, oral cavity, respiratory tract, digestive tract, or other sites of the human body [69]. Differentiating between colonization and acute infection in critically ill patients is often challenging [70].

Regarding antibiotic therapy, most patients received combinations of at least two antibiotics, while only 13.1% received monotherapy, which is consistent with other studies [16,20,71]. The preferred antibiotics for the treatment of VAP were combinations of different classes of antibiotics: carbapenems (26%), third-generation cephalosporins (24.9%), fluoroquinolones (13.9%), and aminoglycosides (6.9%), which is consistent with other reports [72,73]. The antibiograms performed on the patients’ pathological secretions revealed antibiotic resistance. For carbapenem resistance, 52% of the resistant bacteria were *Acinetobacter* spp., followed by *Pseudomonas* spp. at 26% and *Klebsiella* spp. at 23%. *Acinetobacter* spp. was also the most resistant bacteria to fluoroquinolones and aminoglycosides, accounting for 48% of the resistant strains for both antibiotics. Resistance to third-generation cephalosporins was primarily attributed to *Pseudomonas* spp., representing 67% of the resistant species. Souza et al. reported resistance to carbapenems of 28% among *Pseudomonas aeruginosa* strains [74], whereas Gondal et al. found that 80.7% of the *Pseudomonas* spp. strains studied were phenotypically carbapenemase-producing, while 19.3% were non-carbapenemase-producing in the development of the MDR pathogen [75]. In contrast, Ramadan et al. reported that 86.3% of the isolated *Klebsiella* spp. produced extended-spectrum beta-lactamase and 72% were carbapenem-resistant [72].

The relationship between the length of hospitalization and the emergence of MDR pathogens exhibited high sensitivity and specificity, with patients hospitalized for more than 11.5 days showing a significant association (*p* = 0.001; Figure 4). Alnimr (2023) found that a duration of 5 days of ICU admission allowed for the development of MDR pathogens [20], while Torres et al. (2017) reported that an ICU stay of more than 7 days is an independent risk factor for the development of MDR strains [34]. Additionally, MV exceeding 196 h was positively correlated with the development of MDR pathogens (*p* = 0.002) in our study (Figure 4). We did not find clear data in the literature regarding this correlation, so further studies are needed for clarification.

We must also emphasize the important role of ICU scores, such as APACHE or SOFA, in evaluating the severity and mortality risk of critically ill patients. The CPIS in our study correlated with the development of MDR pathogens. Gursel et al. reported a significant association between the mean value of APACHE II, SOFA, and CPIS scores with VAP and mortality [76]. Patient mortality was also associated with both a prolonged ICU stay and an extended duration of MV [77,78].

Anxiety related to COVID-19 may have contributed to slightly poorer care, which could be linked to overcrowding in the ward with critically ventilated patients. Additionally, infection prevention and control measures may have been compromised because of over-staffing, potentially explaining the increased risk of nosocomial infections.

These elements could have significantly impacted the incidence of VAP and the increased risk of death. Finally, the impact of VAP will remain an open issue that requires careful monitoring both locally and globally.

## 4. Materials and Methods

### 4.1. Study Design

We conducted a study to examine the frequency and characteristics of VAP in ICU patients with and without COVID-19 infection at the Mureș Clinical County Hospital, Târgu Mureș, Romania. The study was observational, single-center, and retrospective. It involved patients admitted to the ICU of the Clinical County Hospital Mureș from 1 April 2021 to 1 April 2023.

Adult patients who received MV for at least 48 h and were diagnosed with VAP, whether early-onset (within 4 days) or late-onset (≥5 days) after starting MV were included in the study [34,79].

Patient data, including demographic characteristics, COVID-19 diagnosis, other comorbidities, blood tests, microbiological cultures of pathogen samples, and other relevant information, were extracted from their medical records.

### 4.2. Participants and Procedure

Out of the 912 patients who underwent MV in the ICU of the Mureș County Clinical Hospital during the study period, 122 (13.37%) met the inclusion and exclusion criteria. The inclusion criteria were as follows: patients over 18 years who were hospitalized in the ICU of the Mureș Clinical County Hospital; patients who underwent MV (IMV or NIV) for over 48 h; clinical suspicion, new or progressive and persistent radiologic infiltrates; and positive microbiological cultures from respiratory tract products. Exclusion criteria included the following: patients under the age of 18; and patients who presented imaging, microbiological, or laboratory criteria suggestive of respiratory tract infection less than 48 h after initiation of MV.

The collected data from the patients’ medical records included laboratory tests comprising leukocyte count, neutrophils count, lymphocyte count, monocyte count, eosinophil count, basophil count, platelet count, fibrinogen values, CPR value, and procalcitonin values taken at admission, 24, and 72 h post admission. Imaging investigations such as chest X-ray and CT scans were also documented and considered positive if there was an area of condensation, opacity, or infiltration. Bacteriological examinations were performed from ETA samples or sputum, and antibiograms identified sensitivity and resistance to antibiotics.

In addition, we included clinical scores, such as the Carmeli, APACHE, and SOFA scores, and SARS-CoV-2 test results, the type of ventilation, and the number of hours spent under ventilation.

### 4.3. Diagnosis of VAP

The diagnosis of VAP is based on clinical suspicion alongside new, progressive, and persistent radiological infiltrates. In addition, at least two of the following criteria must be met: a body temperature exceeding 38 °C or falling below 36 °C, a blood cell count showing leukocytosis of over 10,000 cells/mL or leukopenia of fewer than 5000 cells/mL, the presence of purulent tracheal secretions, positive microbiological cultures from lower respiratory tract samples, and a deterioration of gas exchange [17,32,34,79].

The diagnosis of pulmonary fungal infection was confirmed by isolating fungal agents from bronchial aspirates. Their presence is linked to a higher risk of developing VAP, especially when accompanied by imaging changes, tracheobronchial secretions, inflammatory changes, and no concurrent isolation of other bacterial agents [28]. None of the patients underwent lung biopsies.

### 4.4. Statistical Analysis

Statistical analysis of the collected data was conducted using IBM SPSS version 26 (IBM Corp., Armonk, NY, USA), while data manipulation was performed using Microsoft Excel 365 (Microsoft Corp., Redmond, WA, USA). The significance level for the *p*-value was set to 0.05, and the confidence interval (CI) was 95%. The analysis included descriptive statistics, such as mean, median, standard deviation, and interquartile range, and inferential statistics consisting of normality tests (Shapiro–Wilk), tests for comparing the central tendency: Student’s *t*-test for an unpaired Gaussian distribution and the Mann–Whitney test for a non-Gaussian distribution. For comparing contingency tables, we used the chi-square test or Fisher exact test. The receiver operating characteristic (ROC) curve analysis was used to test the predictive power and to determine the cut-off values of the CPIS, hospital stay, and the number of hours spent under MV in developing MDR bacteria.

### 4.5. Definition of MDR/XDR/PDR/DTR

MDR was defined as acquired nonsusceptibility to at least one agent from three or more classes of antimicrobials; extensive drug resistance (XDR) was defined as insensitivity to at least one agent from two antimicrobial categories (bacteria are sensitive to only one or two antimicrobial categories); pan-drug resistance (PDR) as a nonsusceptibility to all agents in all antimicrobial categories and difficult-to-treat resistance (DTR) has been proposed for nonsusceptibility to all first-line agents showing high efficacy and low toxicity (i.e., carbapenems, β-lactam-β-lactamase inhibitor combinations, and fluoroquinolones) [80,81].

## 5. Study Limitations

Our study has several limitations. The study included a total of 122 patients, which is relatively small because it was a monocentric study conducted at a single hospital with both medical and surgical specialties supported by the ICU department. Notably, only 13.4% of the total number of ventilated patients met the inclusion criteria for the study based on the definition of VAP. Such a low inclusion rate makes these findings hard to generalize. However, we believe that these patients diagnosed with VAP over just 2 years represent a major public health issue, not only in our hospital but also in Romania, Europe, and globally. Given the high mortality associated with this condition, we considered it useful to report our data.

The microbiological diagnosis was based on bronchial aspirates, and we could not specify how many of these samples were obtained through bronchoalveolar lavage. Another limitation of our study is the lack of a lung biopsy in cases of VAP caused by *Candida* spp.

## 6. Conclusions

The most common pathogens isolated in our VAP patients were *Acinetobacter* spp., *Pseudomonas aeruginosa*, *Klebsiella pneumoniae*, and *Staphylococcus aureus*.

Resistance to the major classes of antibiotics has reached alarming levels. Therefore, continuous surveillance of these pathogens is essential.

The presence of COVID-19 was associated with a longer duration of mechanical ventilation and a higher risk of death. COVID-19 patients who underwent invasive mechanical ventilation (IMV) had a greater risk of death compared to those who received non-invasive ventilation (NIV). Additionally, the length of hospitalization and the total hours of ventilation were linked to the development of multidrug-resistant (MDR) pathogens and an increased risk of death in both patient groups.

The incidence of MDR bacteria was notably higher among patients, with non-COVID-19 patients showing a greater relative frequency of antimicrobial-resistant *Acinetobacter baumannii*. All strains of MDR *Acinetobacter* spp. and *Klebsiella pneumoniae* were resistant to carbapenems in both COVID-19-positive and negative patients. Interestingly, the resistance of Pseudomonas aeruginosa MDR strains to carbapenems was found to be more prevalent in COVID-19-negative patients.

Lastly, the CPIS was significantly correlated with the development of MDR pathogens in both groups of patients.

Infection prevention and antibiotic administration policies must be adapted to each health facility based on the local resistance profile. Our study highlights the need to identify and understand the profile of local infections to promptly and appropriately manage them and to limit and control the spread of MDR cases.

## Figures and Tables

**Table 1:** Attrition table regarding the repartition of the patients admitted to Clinical County Mures Hospital in the investigated interval.

Attrition Criteria	N
Initial population: patients admitted to Mures County Clinical Hospital from 1 April 2021 to 1 April 2023.	11,648
Patients hospitalized in departments other than the ICU at Mures County Clinical Hospital.	7111
Patients who were hospitalized in post-anesthesia care units.	1196
Patients admitted to the ICU during this interval.	3341
Patients who required MV in the ICU.	912
Final eligible population.	122

**Table 2:** Demographic and clinical characteristics.

Sample Characteristics	All Patients N = 122, n (%)	COVID Positive Patients N = 53, n (%)	COVID Negative Patients N = 69, n (%)	*p* Value
Sex				0.553
Male	75 (61.5)	31 (58.5)	44 (63.8)	
Female	47 (38.5)	22 (41.5)	25 (36.2)	
Living environment				0.422
Rural	74 (60.7)	30 (56.6)	44 (63.8)	
Urban	48 (39.3)	23 (43.4)	25 (36.2)	
Carmeli score				0.005
1	3 (2.5)	2 (3.8)	1 (1.4)	
2	55 (45)	32 (60.4)	25 (33.3)	
3	64 (52.5)	19 (35.8)	45 (65.2)	
Comorbidities				0.155
Cardiovascular				
Yes	97 (79.5)	39 (73.5)	58 (84)	
No	25 (20.5)	14 (26.5)	11 (16)	
Renal				0.323
Yes	38 (31.1)	14 (26.4)	24 (34.7)	
No	84 (68.9)	39 (73.6)	45 (65.3)	
Pulmonary				0.089
Yes	40 (32.8)	13 (24.5)	27 (39.1)	
No	82 (67.2)	40 (75.5)	42 (60.9)	
Oncologic				0.512
Yes	24 (19.7)	9 (16.98)	15 (21.7)	
No	98 (80.3)	44 (82.02)	54 (78.3)	
Digestive				0.08
Yes	50 (40.9)	17 (32)	33 (47.8)	
No	72 (59.1)	36 (68)	36 (52.2)	
Psychiatric				0.952 *
Yes	17 (13.9)	8 (15)	9 (13)	
No	105 (86.1)	45 (85)	60 (87)	
Diabetes				0.063 *
Yes	27 (22.1)	7 (13.2)	20 (28.9)	
No	95 (77.9)	46 (86.8)	49 (71.1)	
Obesity				0.376 *
Yes	24 (19.7)	8 (15)	16 (23.1)	
No	98 (80.3)	45 (85)	53 (76,9)	
Number of comorbidities				
0	2 (1.6)	2 (3.8)	0 (0)	<0.001 ^†^
1	23 (18.9)	15 (28.3)	8 (11.6)	
2	29 (23.8)	17 (32.1)	12 (17.4)	
3	43 (35.2)	13 (24.5)	30 (43.5)	
4	19 (15.6)	4 (7.5)	15 (21.7)	
5	5 (4.1)	1 (1.9)	4 (5.8)	
6	1 (0.8)	1 (1.9)	0 (0)	
Median (IQR)	3 (1)	2 (2)	3 (2)	
Invasive devices				
Central venous catheter				
Yes	110 (90.2)	52 (98.1)	58 (84)	0.012 ^#^
No	12 (9.8)	1 (1.9)	11 (16)	
Urinary catheterization				
Yes	73 (59.8)	16 (30.2)	57 (82.6)	<0.001
No	49 (40.2)	37 (69.8)	12 (17.4)	
Surgical intervention during the last 30 days				
No	101 (82.8)	48 (90.6)	53 (76.8)	0.055 ^#^
Yes	21 (17.2)	5 (9.4)	16 (23.2)	
APACHE score				
Median (IQR)	22 (10)	24 (10)	17 (11)	0.065 ^†^
SOFA score				
Median (IQR)	10 (5)	10 (4.25)	8 (6.5)	0.360 ^†^
Conventional radiology result				
Persistent infiltrates	52 (49)	27 (40)	34 (55.7)	0.109
Evolutive radiological changes	54 (51)	18 (60)	27 (44.3)	
Computed tomography result				
Persistent infiltrates	18 (48.6)	5 (29.4)	13 (65)	0.049 *
Evolutive radiological changes	19 (51.4)	12 (70.6)	7 (35)	
VAP bacteriologic diagnosis				
No	40 (32.8)	20 (37.8)	20 (29)	0.307
Yes	82 (67.2)	33 (62.2)	49 (71)	

N—number of patients, IQR—interquartile range. The contingency tables were analyzed using the chi-square test, chi-square test with Pearson correction (marked with *), or Fisher’s exact test (marked with #). The number of comorbidities, APACHE score, and SOFA score were compared using the Mann–Whitney U test (marked with ^†^).

**Table 3:** Laboratory data.

	COVID-19 Positive Patients Mean ± SD/Median (Q1–Q3)	COVID-19 Negative Patients Mean ± SD/Median (Q1–Q3)	*p*-Value
Age (years)	70.04 ± 11.48	66.58 ± 11.79	0.107 ^†^
Intensive care stay (days)	12 (8–18.5)	10 (7–15)	0.242
Duration of ventilation (hours)	245 (134–404.5)	152 (86–240)	0.001 *
Neutrophil (×10^3^/µL) at admission	9.09 (5.89–14.04)	8.74 (6.43–13.79)	0.938
Neutrophil (×10^3^/µL) at 48 h	11.74 (7.28–16.99)	9.2 (6.15–13.94)	0.139
Neutrophil (×10^3^/µL) at 72 h	12.60 (8.54–19.15)	9.28 (6.61–13.75)	0.006 *
Lymphocytes (×10^3^/µL) at admission	0.55 (0.44–0.93)	0.8 (0.47–1.32)	0.073
Lymphocytes (×10^3^/µL) at 48 h	0.59 (0.38–0.89)	0.88 (0.41–1.16)	0.083
Lymphocytes (×10^3^/µL) at 72 h	0.46 (0.37–0.74)	0.88 (0.53–1.39)	<0.001 *
Monocytes (×10^3^/µL) at admission	0.41 (0.26–0.72)	0.59 (0.38–0.9)	0.039 *
Monocytes (×10^3^/µL) at 48 h	0.45 (0.28–0.69)	0.56 (0.29–0.84)	0.292
Monocytes (×10^3^/µL) at 72 h	0.41 (0.26–0.81)	0.57 (0.37–0.97)	0.006 *
Eosinophils (×10^3^/µL) at admission	0.02 (0–0.09)	0.02 (0–0.08)	0.935
Eosinophils (×10^3^/µL) at 48 h	0.01 (0–0.04)	0.04 (0.01–0.15)	0.016 *
Eosinophils (×10^3^/µL) at 72 h	0.01 (0–0.08)	0.08 (0.01–0.26)	0.001 *
LDH (U/L)	456 (274.5–709)	298 (224.5–387.75)	0.007 *
Neutrophil/lymphocyte ratio at admission	15.11 (7.72–25.66)	11.80 (7.18–23.70)	0.311
Neutrophil/lymphocyte ratio at 48 h	17.80 (10.11–35.37)	12.02 (6.26–20.78)	0.016 *
Neutrophil/lymphocyte ratio at 72 h	24.31 (11.74–44.23)	10.13 (5.63–19.11)	<0.001 *

The *p*-values were calculated using the Mann–Whitney test (they had a non-Gaussian distribution), except those marked with ^†^, where the Student’s *t*-test for unpaired data was used (they had a Gaussian distribution). The *p*-values marked with * had a value below 0.05. Mean ± SD or Median (Q1–Q3) was used according to the Gaussian distribution of the data. SD = standard deviation, Q1 = first quartile, Q3 = third quartile, LDH = lactate dehydrogenase.

**Table 4:** Pathogens involved in VAP.

Pathogen Agent	COVID-19 Positive Patients’ Relative Frequency	COVID-19 Negative Patients’ Relative Frequency
*Acinetobacter* spp.	39.29%	32.34%
*Baumannii*	10.71%	2.94%
*Baumannii* MDR	17.86%	29.41%
*Jejuni* MDR	10.71%	0.00%
*Pseudomonas* spp.	21.43%	11.76%
*Aeruginosa*	7.14%	5.88%
*Aeruginosa* MDR	14.29%	11.76%
*Klebsiella* spp.	14.29%	11.76%
*Pneumoniae*	3.57%	2.94%
*Pneumoniae* MDR	10.71%	8.82%
*Stafilococus aureus* spp.	7.14%	8.82%
MSSA	3.57%	5.88%
MRSA	3.57%	2.94%
*Stenotrophomonas* *maltophilia*	3.57%	5.88%
*Escherichia* spp.	0.00%	0.00%
*Coli*	0.00%	2.94%
*Coli* MDR	0.00%	2.94%
*Citrobacter Freundii*	3.57%	0.00%
*Corymenacterium Striatum*	0.00%	2.94%
*Morganella morganii*	0.00%	2.94%
*Serratia Marcescens*	0.00%	2.94%
*Candida Albicans*	3.57%	2.94%
*Candida Non-Albicans*	3.57%	2.94%
*Candida Glabrata*	3.57%	0.00%
*Candida Krusei*	0.00%	2.94%

MDR = multi-drug-resistant, MSSA = methicillin-susceptible *Staphylococcus aureus*, MRSA = methicillin-resistant *Staphylococcus aureus*.

**Table 5:** Antimicrobial treatment.

Antibiotics	Relative Frequency
Penicillins	4.6%
Second-generation cephalosporins	0.6%
Third-generation cephalosporins	24.9%
Carbapenems	26%
Aminoglycosides	6.9%
Antifungals	2.9%
Oxazolidinones	9.8%
Fluoroquinolones	13.9%
Polymyxins	10.4%
Type of therapy	
Monotherapy	13.1%
Polytherapy	86.9%

**Table 6:** VAP/MDR relationship.

Variable	Cut-Off	AUC	95% CI	Sensitivity	Specificity	*p*-Value
Multi-drug resistance in all patients						
Days spent in intensive care	11.5	0.708	0.595–0.820	78.6%	63.8%	<0.001
Hours spent ventilated	196	0.695	0.570–0.820	78.6%	56.4%	0.002
CPIS	5	0.854	0.786–0.921	89.3%	74.5%	<0.001
Multi-drug resistance in COVID-positive patients						
Days spent in intensive care	11.5	0.618	0.453–0.783	78.6%	56.4%	0.193
Hours spent ventilated	261.5	0.604	0.433–0.776	71.4%	61.5%	0.250
CPIS	5	0.905	0.825–0.984	100%	79.5%	<0.001
Multi-drug resistance in COVID-19-negative patients						
Hours spent in intensive care	11.5	0.773	0.621–0.925	78.6%	69.1%	0.002
Days spent ventilated	196	0.731	0.533–0.929	78.6%	72.7%	0.008
CPIS	5	0.800	0.692–0.908	78.6%	70.9%	0.001

**Table 7:** Mortality analysis.

Variable	Discharged Dead Median (Q1–Q3)	Discharged Alive Median (Q1–Q3)	*p*-Value
Intensive care stay (days)	12.00 (8.00–18.00)	9.00 (6.75–15.00)	0.017 *
Duration of ventilation (hours)	222.50 (138.50–396.00)	122.50 (90.00–232.50)	0.001 *
Leucocytes (×10^3^/µL) at admission	10.85 (7.73–16.41)	9.44 (6.85–13.44)	0.122
Leucocytes (×10^3^/µL) at 48 h	13.37 (9.84–20.81)	9.07 (6.49–11.05)	<0.001 *
Leucocytes (×10^3^/µL) at 72 h	14.15 (10.42–20.20)	10.65 (7.04–12.81)	<0.001 *
Neutrophil (×10^3^/µL) at 48 h	12 (8.64–18.83)	7.24 (5.32–9.75)	<0.001 *
Neutrophil (×10^3^/µL) at 72 h	12.61 (8.6–18.44)	8.02 (5.23–10.4)	<0.001 *
Lymphocytes (×10^3^/µL) at 72 h	0.56 (0.37–0.99)	0.94 (0.51–1.49)	0.004 *
Eosinophils (×10^3^/µL) at 48 h	0.02 (0.00–0.07)	0.04 (0.01–0.16)	0.100
Eosinophils (×10^3^/µL) at 72 h	0.02 (0.01–0.11)	0.10 (0.02–0.31)	0.001 *
Basophils (×10^3^/µL) at 72 h	0.00 (0.00–0.01)	0.01 (0.00–0.02)	0.004 *
CRP (mg/dL) at 48 h	9.74 (5.39–15.87)	4.82 (2.42–10.84)	0.011 *
LDH (U/L)	387.50 (240.25–720.25)	313.00 (230.00–383.00)	0.045 *
COVID-19			
Yes	43 (35.3%)	10 (8.2%)	0.002 *
No	37 (30.3%)	32 (26.2%)	
Type of ventilation			
NIV	15 (12.3%)	29 (23.8%)	<0.001 *
IMV	46 (37.7%)	8 (6.6%)	
Mixed	19 (15.5%)	5 (4.1%)	
Chest radiography			
Evolutive radiological changes	42 (39.7%)	12 (11.3%)	0.003 *
Persistent infiltrates	26 (24.5%)	26 (24.5%)	
Chest CT			
Evolutive radiological changes	16 (43.2%)	3 (8.2%)	0.007 *^,†^
Persistent infiltrates	7 (18.9%)	11 (29.7%)	

The *p*-values were calculated using the Mann–Whitney test (if they had a non-Gaussian distribution) or with chi-square test or Fisher exact test (marked with ^†^) if they were presented in a contingency table format. The *p*-values marked with * had a value below 0.05. SD = standard deviation, LDH = lactate dehydrogenase.

## Data Availability

The data will be shared upon request.

## Abbreviations

APACHE	Acute Physiology and Chronic Health Evaluation
AUC	Areas under the curve
COVID-19	Coronavirus disease 2019
CPIS	Clinical Pulmonary Infection Score
CRP	C reactive protein
DTR	Difficult-to-treat resistance
ETA	Endotracheal aspiration
ETT	Endotracheal tube intubation
HAP	Hospital acquired pneumonia
ICU	Intensive care unit
IMV	Invasive mechanical ventilation
LDH	Lactate dehydrogenase
NIV	Noninvasive mechanical ventilation
MDR	Multiple drug resistance
MRSA	Methicillin-resistant *Staphylococcus aureus*
MSSA	Methicillin-sensitive *Staphylococcus aureus*
MV	Mechanical MRSA
ROC	Receiver operating characteristic curves
PACU	Post-anesthesia Intermediate Intensive Care Unit
PCT	Procalcitonin
PCR	C-reactive protein
PDR	Pan-drug resistance
SARS-CoV-2	Severe acute respiratory syndrome coronavirus 2
SD	Standard Deviation
SOFA	Sequential Organ Failure Assessment
VAP	Ventilator-associated pneumonia
Q1	First quartile
Q3	Third quartile
XDR	Extensive drug resistance
